# The Morphology and Microstructure of Oxide Scale Grown on Austenitic Steel during Steam Oxidation at 700 °C for 500 h

**DOI:** 10.3390/ma14143821

**Published:** 2021-07-08

**Authors:** Bogdan Rutkowski, Krzysztof Baran, Remigiusz Błoniarz, Tomasz Kozieł

**Affiliations:** Faculty of Metals Engineering and Industrial Computer Science, AGH University of Science and Technology, al. A. Mickiewicza 30, 30-059 Kraków, Poland; kris@agh.edu.pl (K.B.); bloniarz@agh.edu.pl (R.B.); tkoziel@agh.edu.pl (T.K.)

**Keywords:** steam oxidation, STEM, SEM, austenitic steel, SAVE25

## Abstract

The modern power generation industry needs materials able to withstand severe conditions, such as high temperatures, steam pressure, and an aggressive environment, to create more electric power out of a decreasing amount of fuel. Therefore, new metallic materials are continuously being developed. In order to gain knowledge about modern materials, the investigation of high Cr and Ni austenitic steel oxidized in 100% steam at 700 °C for 500 h was performed. The morphology, the phase composition, and the chemical composition of the oxidation products were investigated through methods of advanced electron microscopy techniques. Moreover, precipitates present in bulk material were identified. The material developed a continuous and complex oxide scale, consisting of Fe_2_O_3_, Cr_2_O_3,_ and spinel phases. Very fine MX, fine ε-Cu, and M_23_C_6_ precipitates were found in the bulk material. The creation of iron oxide is induced due to the coarse grain size of the material. Cr_2_O_3_ forms due to the internal oxidation process.

## 1. Introduction

Strong economic and population growth is incessantly leading to an increase in energy consumption, which may rise nearly 50% between 2020 and 2050. Therefore, due to the easy access to coal deposits, coal power plants remain an important and relatively stable source of energy [1,2]. Even if the renewable energy production starts soaring, it is necessary to use coal-fired power plants to support them [3]. Unfortunately, traditional methods of coal combustion emit a huge amount of greenhouse gases and toxic airborne emissions relative to other power generation options [4]. At present, however, politicians and ecological organizations are set on decreasing CO_2_ emissions. The solution is to significantly improve the efficiency of power plants by increasing the temperature and pressure of steam [3,5]. Currently, most coal-fired power plants work up to a temperature of 600 °C, achieving an efficiency of up to 40%. Increasing the working temperature to 700 °C will allow them to gain efficiency to about 44%, which will enable a reduction in a considerable quantity of CO_2_ emission [6]. However, increasing the operating temperature significantly accelerates the oxidation rate of materials. Worthy of mention is that the materials developed to work as steam superheaters have to withstand severe conditions of two aggressive environments that cause oxidation. The first of them is the hot and high-pressured steam, flowing inside of the tubing. The second one is the flue gas, formed as a result of fuel (coal, lignite, and gas) combustion, which can be highly degrading to the material if the fuel consists of aggressive impurities (e.g., S). Higher steam temperatures in connection with increased pressure, induces not only the higher plastic deformation due to the creep, but might also cause severe oxidation damage. Various steam and air/water vapor oxidation studies have been performed, which allows for further materials development to decrease the oxidation rate of the alloys [7]. In a perfect situation, steel would develop a thin and protective chromia scale, which would further protect the alloy. Unfortunately, Cr_2_O_3_ coexists with various oxides (e.g., Fe_2_O_3_, Fe_3_O_4_, and Fe_2_CrO_4_ spinel), which are formed due to the severe operating conditions. Such an amount of various oxides often leads to spallation or exfoliation due to the different thermal expansion coefficients of coexisting oxides. This may result either in higher oxidation rates of steels or even as a strong contribution to the accelerated erosion of the steam turbine components. In very extreme conditions of air and vapor mixture, Cr may even evaporate, due to the formation of volatile hydroxides. This leads to failure due to breakaway corrosion [7]. Therefore, it becomes necessary to implement alloys with superior oxidation resistance under steam and flue gas environments at high temperature [8,9,10,11].

Ni-based alloys are great at fulfilling these requirements in boiler applications, but due to the very high price of nickel, their usage may be economically unjustified. Austenitic steels seem to be an excellent alternative due to their lower price, while maintaining sufficient properties [6,12]. One of the possibilities is the SAVE 25 (23Cr-18Ni-3Cu-1.5W-Nb-N) [13,14,15] stainless steel, developed by Sumitomo Metal Industries for the superheater/reheater section in fossil power plants. SAVE 25 is characterized by a higher creep rupture strength than other similar materials, and has high hot corrosion and steam oxidation resistance [14]. The desirable properties of this alloy are a result of the high content of chromium (approx. 23 wt.%) and the addition of such elements as 1.5% W for solid solution strengthening, 3% Cu for precipitation strengthening by the fine dispersed Ɛ-Cu phase, and 0.2% N for austenite stabilization [15,16]. Tungsten, in addition, plays an important role in alloy strengthening, since both, coarsening of the strengthening precipitates and the precipitation of the sigma phase are suppressed. Therefore, a secondary creep regime is extended [14]. Moreover, the addition of ~3% of Cu has a positive impact on the creep strength of the 23Cr-15.5Ni-2.5W-0.2N alloy. The time to rupture increases from ~1350 to ~2200 h (about 60%) under the stress of 157 MPa at 700 °C [14]. Such a combination of strengthening mechanisms predestinates the SAVE 25 alloy to operate at high temperature. Until now, SAVE 25 has rarely been the subject of research, and there is little information about actual applications. Alarming research results were published in the report “Coal Ash Corrosion Resistant Materials Testing” [17], in which SAVE 25 had the highest rate of metal loss from among twelve investigated candidates for coal power plant application. Moreover, the oxidation of the tube was very uneven. The wall thickness in the most severely degraded area was around 5.5 times smaller than in the thickest part of the tube. Additionally, a pinhole leak was also reported [17]. Speculations were also quoted that copper might cause decohesion between the scale and underlying metal, or inhibit the formation of the protective chromium oxide scale [17,18]. Although the alloy has a suitable chemical composition in order to withstand severe conditions, its oxidation resistance seems to be insufficient. Therefore, the currently reported results might give a better insight into material behavior at high temperature.

In the present work, a steam oxidation process on 23Cr-18Ni-3Cu-1.5W-Nb-N steel was carried out at 700 °C for 500 h in a test rig specially designed for a high temperature oxidation test. The microstructure and chemical composition were investigated using modern electron microscopy techniques.

## 2. Materials and Methods

**Figure 1:**
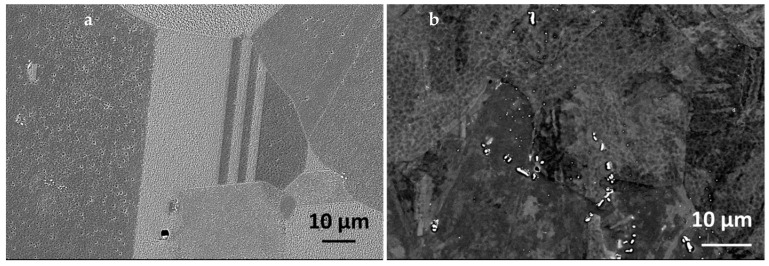
Microstructure of the as-received 23Cr-18Ni-3Cu-1.5W-Nb-N steel: (**a**) Transverse cross-section, (**b**) Longitudinal cross-section.

The material used in this study was stainless steel, smelted in-house using the Arc Melter AM (Edmund Böhler, GmbH, Bodelshausen, Germany). The investigated alloy was synthesized under the Ti-gettered argon atmosphere by an arc melting of Ni-based HR6W alloy, Fe-Mn and Fe-C pre-alloys, Fe Armco and high purity elements (Cr, Ni, Cu, Nb). In order to ensure the homogeneity of the chemical composition, the ingot was re-melted five times. The obtained ingot with a thickness of approx. 10 mm was heated (40 min, at 1200 °C) and further hot-rolled using a 4-high laboratory rolling mill. The deformation process was conducted with interpass reheating every 2 passes. This meant that dynamic continuous recrystallization was achieved. Deformation at high temperature as well as a short duration (5 min) annealing after deformation and cooling in the air assured that carbides and nitrides precipitated in an equilibrium state upon cooling (hence, a strain induced precipitation mode was not activated). The final specimen with a 3 mm thickness was free of the structural effects of the deformation. Such treatment led to obtaining an alloy with the chemical composition presented in Table 1 and the typical austenitic microstructure, with the grain size of 44.4 (±11.5) µm, containing primary NbX and secondary precipitates (Figure 1).

To prepare samples intended for further oxidation, coupons 10 mm × 10 mm wide and about 2 mm thick were cut. The next stage was the proper surface preparation for the high temperature oxidation test. For this purpose, the tested surfaces were gradually ground on the abrasive papers, with gradations changed from 400 to 4000. The surface, prepared in this way, was successively polished to obtain a mirror-like surface by using SiO_2_ suspension. The state of the sample surface has a great impact on oxidation test results for various alloys [19,20]. Samples prepared in this way were subject to high temperature oxidation in 100% steam in a specially designed, closed loop test rig PRC 110M/GWP (Czylok, Jastrzębie Zdrój, Poland).

**Figure 2:**
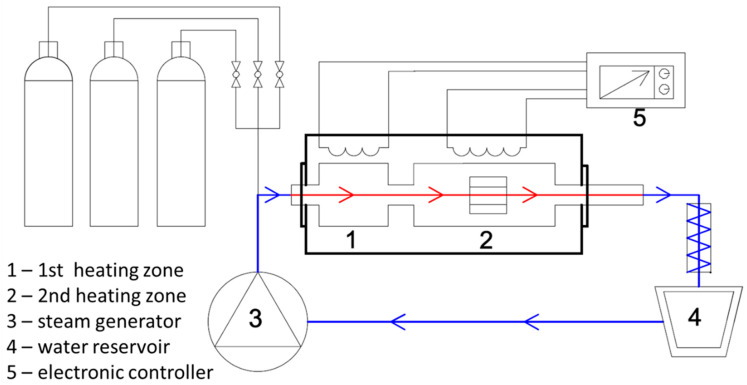
The steam oxidation rig used for the corrosion experiments; the explanation is in the text.

The setup of this test stand (Figure 2) contained a two-zoned tube furnace. The first zone (1) was designed for the pre-heating of the supplied gases. The second zone (2) was a reactor, where samples were heated up to proper temperature undergoing oxidation. Another important element of this test rig was the steam generator (3). What is significant is that the test stand had a closed water loop. With this configuration, previously formed steam, after passing through the reactor, was condensed and went to the water reservoir (4), from where it returned to the steam generator. The entire installation was gas tight, preventing the access of air under atmospheric pressure. This met the steam power plant’s demand of low oxygen/air content [7]. The water used in the process was previously deionized. Due to the relatively short time of oxidation, the pH of the water was not controlled during the oxidation process. The whole process was controlled using a special electronic controller (5). The oxidation test was carried out at 700 °C up to 500 h. The specimen was removed from the chilled furnace after the allotted time. Afterwards, the mass gain was measured, and the specimen was inserted into the furnace and heated up for further oxidation [21]. The mass gain after a certain time was measured with high resolution (1 µg) MYA 5.4.Y Plus microbalance of Radwag (Radom, Poland).

At the beginning, the surfaces of the oxidized samples were examined using the scanning electron microscope (SEM)—Merlin Gemini II of ZEISS (Oberkochen, Germany). Then, in order to prepare the samples for detailed microscopic studies, a nickel coating was applied. It allows for the protection of an oxidized surface from destruction during cutting and further processing. Next, the samples were sequentially embedded in the resin and then grounded on the abrasive papers and polished on the SiO_2_ suspension. The sample preparation process was described in detail previously [21]. Such cross-sections underwent further investigations by the methods of scanning electron microscopy.

Additionally, techniques of analytical (scanning) transmission electron microscopy ((S)TEM) were performed using Titan^3^ G2 60–300, with Cs correction of the illumination system (Thermo Fisher Scientific, Eindhoven, The Netherlands). This allowed us to obtain high resolution (HR) images in the STEM mode, which were further processed to extract crystallographic data from fast Fourier transformed (FFT) images. Such processing is complementary to the conventional selected area electron diffraction (SAED); however, it has an advantage over SAED, since nano-areas (of few nm^2^) can be investigated. Such a technique was used to determine the crystal lattice of the fine carbides, dispersed in the matrix. The high-angle annular dark field (HAADF) technique allowed us to distinguish Cu-rich precipitates among the matrix. High quality energy dispersive X-ray spectroscopy (EDS) elemental maps were possible to obtain due to silicon drift detectors (SDD), which were extremely helpful, especially during the phase analysis of the oxide scale. In order to ensure the highest quality results, the sample for the STEM analysis has to be thin, and its thickness should be uniform in the whole area. Therefore, it was prepared by the focus ion beam (FIB) method, as described previously [21].

## 3. Results and Discussion

The main difference between the oxidation in pure steam and the oxidation in the air and water vapor mixture is the formation of the volatile CrO_2_(OH)_2_ in the latter case, which changes the oxidation mechanism [7]. In the case of the reported approach, the formation of volatile compounds is not foreseen. While the oxidation is in pure steam, the partial pressure of the oxygen might be decreased, due to the formation of H_2_ as a result of the metal surface reaction with steam, which might suppress the hematite formation and decrease the growth of the Fe_3_O_4_ and spinel crystals [22,23].

**Figure 3:**
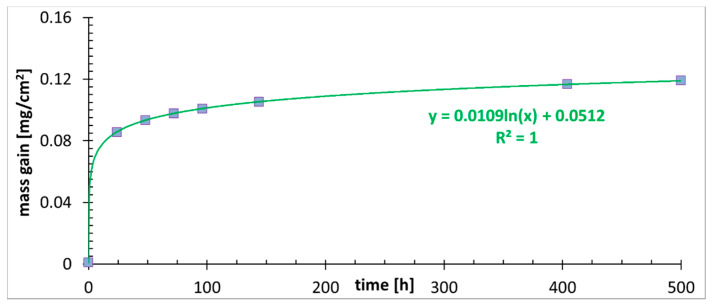
The mass gain measurement at 700 °C up to 500 h.

The mass gain measurement is shown in Figure 3. At the beginning of the oxidation, for the first 20–25 h, the oxidation rate is very fast, and the mass gain is the most pronounced. Therefore, oxides form, although the created layer is discontinuous. After a longer time, the process becomes stable, and the logarithmic law is in charge of the oxidation [24]. It is the transient regime between linear and parabolic law [25]. Since the oxide scale is quite thin and the diffusion path of the oxidant is relatively short, the oxide scale growth is controlled by the surface reaction. When the oxide scale becomes thick enough, the controlling mechanism might change to parabolic one [25]. In that case, the ion diffusion path will be long enough to make the diffusion the slowest, controlling the oxidation, process. No pronounced signs of spallation are detected from the mass gain measurement, and measuring errors are low. On the basis of the obtained plot, it can be concluded that the developed oxide scale is protective. It is a decent result in comparison to the coarse grained TP347H steel, which in the steam reached the mass gain of around 0.11 mg/cm^2^ after 200 h at 650 °C and exhibited breakaway damage, with a weight gain of 3 mg/cm^2^ after around 170 h at 700 °C [26]. Interestingly, similar oxidation kinetics to the one described here were reported for fine grained (~8 µm) 304 steel, modified with Cu and oxidized up to 500 h in atmosphere of air with 20% of steam [27], whereas the coarse grained (~30 µm) material exhibited not only a seven times higher mass gain, but oxidation that was governed by the parabolic kinetic.

**Figure 4:**
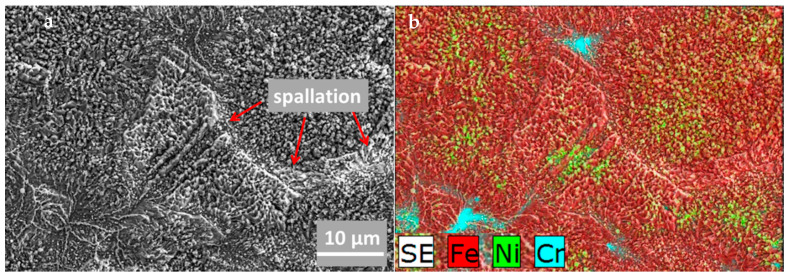
The surface morphology of 23Cr-18Ni-3Cu-1.5W-Nb-N steel, oxidized at 700 °C for 500 h in steam: (**a**) The SEM-SE image; (**b**) SEM-EDS the combined elemental map of the selected elements.

The surface morphology of 23Cr-18Ni-3Cu-1.5W-Nb-N steel after oxidation is presented in Figure 4. It is characterized by the occurrence of slight spallation of the oxide scale, evidenced in Figure 4a. The edges of the damaged area (marked with red arrows) are neither sharp nor clean, which determines the nature of the spallation and indicates its occurrence at high temperature while the oxidation process occurs. This type of spallation is the result of the growth stresses located within oxides, leading to a rapid increase in the oxidation rate [28,29]. The spallation effects while the isothermal oxidation of the austenitic alloy was at 900 and 950 °C were demonstrated with the thermogravimetric method [30]. What is worth mentioning is that the first spallation effects at the lower temperature were noticed after a longer exposure time in comparison to the higher temperature experiments. Our previous investigations on austenitic heat resistant steel [21] demonstrated very different behavior—spallation occurred while cooling down from the oxidation temperature. According to Galerie et al. [31], the presence of Nb contributes to spallation. An EDS microanalysis of the observed surface (Figure 4b) showed that the outer scale surface was enriched with Fe, with some traces of Cr and Ni rich oxides.

**Figure 5:**
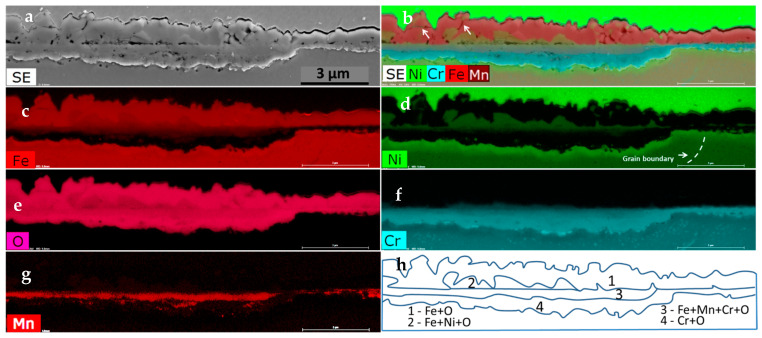
The microstructure of the cross-section of 23Cr-18Ni-3Cu-1.5W-Nb-N steel, oxidized at 700 °C for 500 h in steam: (**a**) The SEM image, the secondary electrons (SE); (**b**) The compositional image showing 4 layers; (**c**–**g**) The SEM-EDS elemental maps of the selected elements; (**h**) A schematic diagram of the grown layers.

Examples of the changes after steam exposure are visible under SEM on the cross-section (Figure 5a). The oxidation layer that grew after 500 h is relatively thin and continuous. Two layers can be observed. The inner layer is characterized by some porosity, while the outer layer has few single cracks visible. Some discontinuity is visible between the above mentioned areas. The average thickness of the oxide layer, measured at the length of 0.3 mm, is 4.4 ± 0.67 μm with the variance of 0.46 μm^2^. The further SEM-EDS elemental composition analysis of the formed scale allowed us to distinguish four layers, as indicated in Figure 5h. Right above the substrate, a continuous chromium oxide layer (#4) is formed. Above it, in addition to Cr, Mn and Fe were also detected (#3). Next, the upper layer (#2) consists of Ni, Fe, and O. What is important is that this layer is characterized by the presence of the cracks mentioned earlier. The outermost layer (#1) contains a substantial amount of Fe and O. It is worth mentioning that, according to Figure 4b and Figure 5b, this iron oxide layer undergoes spallation (cracks are indicated with the white arrows in Figure 5b). Neither cracks nor pores were observed in the chromium oxide layer, which is very important to assure proper oxidation resistance.

**Figure 6:**
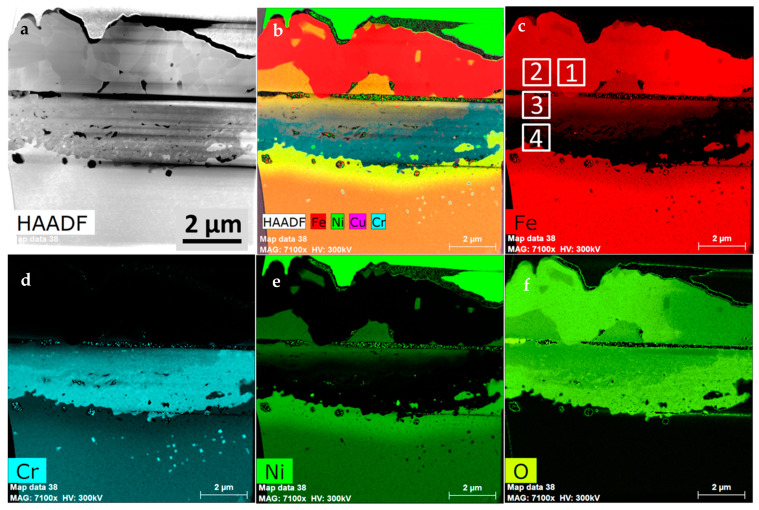
The microstructure of the cross-section of 23Cr-18Ni-3Cu-1.5W-Nb-N steel oxidized in steam at 700 °C for 500 h: (**a**) The STEM-HAADF image; (**b**) The compositional image of the selected EDS maps, (**c**–**f**) The STEM-EDS maps of the selected elements.

In complementary to the SEM-EDS investigations, the (S)TEM investigation was also performed. This allowed for a more detailed chemical composition analysis, as well as phase identification with the selected area electron diffraction (SAED) and the fast Fourier transformed (FFT) high resolution (HR) STEM images. Figure 6a shows a STEM-HAADF image of the lamella, prepared from the metallographic cross section. Therefore, fine grained Ni containing coating was present at the top of the sample. Underneath, a coarse-grained area is visible. The fine-grained region beneath is the internal oxidation zone. The discontinuous interface between the previously mentioned layers is the effect of the sample preparation. Porosity, however, is visible at the interface between layer #1 and #2 as well as between layer #3 and #4. The STEM-EDS results performed at higher magnification on the TEM sample allowed us to perform a more detailed observation. The Cr_2_O_3_ is the most thermodynamically stable in comparison to the iron oxides. Due to its low dissociation oxygen partial pressure, it should form preferentially even under very low oxygen content [7]. However, the Cr_2_O_3_ was not created first, but rather the Fe_3_O_4_, which is the second most stable of the oxides. It may be present in the oxide scale due to the chemical composition of the alloy. Its appearance above the surface level of the alloy suggests that it was formed by the outward diffusion of Fe. Ni also undergoes the outward diffusion, therefore some amounts of Ni dissolves in the magnetite (Fe_3_O_4_) to form the Fe_2_NiO_4_ spinel. It is worthy of mention that this layer is uncontinuous. The suppression of the magnetite formation can be attributed to the formation of an internal oxidation zone, where the Cr oxidizes to Cr_2_O_3_. As the oxygen partial pressure is higher than necessary to form Fe_2_O_3_, the continuous layer of Fe_2_O_3_ is formed. Fe_2_O_3_ is less stable than Fe_3_O_4_ [7], therefore it forms after the latter. Fe_2_O_3_ is created due to the outward diffusion of iron, even through the Fe-Ni spinel lattice. This assumption is supported by the microstructural observation, where Fe_2_O_3_ (#1) was created above the Fe-Ni spinel (#2). That mechanism also explains the lifting up of the small crystals of the Fe-Ni spinel, encapsulated in the upper parts of the Fe_2_O_3_ layer. Ni is easily soluble in Fe_3_O_4_, which leads to the Fe-Ni spinel formation. A sharp change in the chemical composition is visible between layer #1 and #2, which indicates that Ni is not soluble in Fe_2_O_3_, which might have an influence on the significant lack of integrity of the material (spallation). As previously mentioned, Figure 4 evidences the outermost Fe_2_O_3_ layer spallation, which all in all does not have protective properties. The microstructural investigation also reveals that only the oxide scale created over the bigger grains of the austenite (area on the left from the grain boundary marked in Figure 5d) consists of the Ni-enriched crystals. This the effect of the faster Ni diffusion rate over the bulk Cr flux. Noticeable is the 2–3 times thinner oxide scale in the areas where the grain boundary (marked with dashed line in Figure 5d) is directed towards the surface of the alloy and in which no Ni-enriched coarse crystals were found. This suggests that the oxide scale is thinner, since Cr diffusion through grain boundaries is faster than its bulk diffusion and more Cr is supplied, leading to faster Cr_2_O_3_ formation. It hinders the outward diffusion of Fe and slows down the oxidation rate. This is supported by previous investigations, where grain boundaries near the surface area in Sanicro 25 were drained of Cr much deeper than the range of the Cr-depleted zone occurrence. Moreover, the smaller grain was drained out of the Cr through grain boundaries, whereas the larger grain was still enriched with Cr in almost whole volume [32]. In contrary to the behavior described above, there is a continuous change in the chemical composition in the internal oxidation zone, where the amount of Ni and Fe is decreasing while shortening the distance to the bulk material (Figure 6b,d,e), resulting in the presence of Cr oxide only, since Ni is diffused outward to form the Fe-Ni spinel (#2) (Figure 6c,f). The coarse grains of the two upper layers testify to the rapid oxidation of Fe, while only a small amount of crystals were nucleated and grew. Afterwards, where strong Fe-depletion occurred, the alloy started to oxidize internally due to the inward oxygen diffusion, and a higher number of crystals were created. This indicates that even under a relatively thick layer of Fe_2_O_3_ and spinel, the oxygen partial pressure is sufficient to create Cr_2_O_3_ crystals. Therefore, inward oxygen diffusion through Fe-rich oxides is faster than the outward diffusion of Cr. The slow bulk diffusion of chromium in austenite might be confirmed by the shallow Cr-depleted zone in the coarse-grained material, which is attributed to the large grain size of steel [21,33,34]. At the Cr_2_O_3_/alloy interface, the oxygen partial pressure is too low for further oxidation of Cr. Therefore, it needs time to increase the oxygen partial pressure through the inward oxygen diffusion, necessary for Cr_2_O_3_ formation in the deeper part of the material. The formation of the internal Cr_2_O_3_ layer also impedes the outward diffusion of Ni and Fe [7]. This is reflected within lower oxidation rates over longer time periods and thinner oxide scales. The performed investigation gives evidence of the protective properties of the oxide scale, assured by the Cr_2_O_3_ layer. It was, however, created not by the outward diffusion of Cr ions, but due to internal oxidation by the inward diffusion of oxygen. If the oxide scale is thick enough at the bottom of the uppermost layer (#1), oxygen partial pressure might become sufficiently low. Therefore, the process of the dissociation of the least stable Fe_2_O_3_ can start. It will support the lower sublayers to grow by suppling the oxidant, which might explain the porosity visible between Fe_2_O_3_ (#1) and the spinel crystals (#2). Since Fe_2_O_3_ layer thickness is similar to Cr_2_O_3_ layer thickness, it could be concluded that there was no issue with severe H_2_ formation. In the other case, it would decrease the oxygen partial pressure and cause the dissociation of the least stable Fe_2_O_3_ oxide.

The bulk material beneath the internal oxidation zone exhibits the Fe- and Cr- depleted zones, where the amount of Ni is increased (Figure 6e).

**Figure 7:**
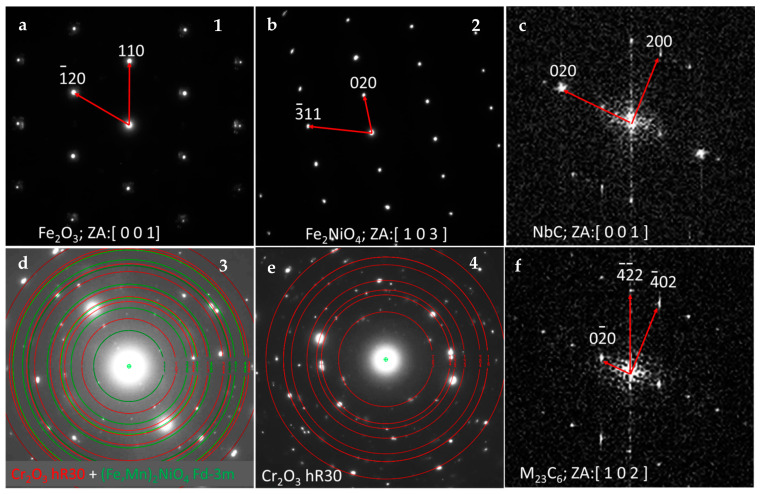
The SAED and FFT results on the areas indicated in Figure 5c,b: (**a**) Fe_2_O_3_, (**b**) Fe_2_NiO_4_, (**c**) NbC, (**d**) Cr_2_O_3_+(Fe,Mn)_2_NiO_4_, (**e**) Cr_2_O_3_, and (**f**) M_23_C_6_.

As was already mentioned, electron diffraction methods in connection with HRSTEM imaging and FFT analysis allows one to distinguish the individual phases occurring across the oxide scale. Selecting from the top, as shown in Figure 7, the outermost part of the oxide scale consists of coarse Fe_2_O_3_ crystals. Beneath, the coarse grains with the lattice identified as Fe_2_NiO_4_ were found. Underneath, the mixture of (Fe, Mn)_2_NiO_4_ and Cr_2_O_3_ was detected, whereas the innermost layer was identified as Cr_2_O_3_. Both innermost layers were fine grained. This situation is slightly different from the case reported earlier [21], where the crystals of the outermost layer were much larger than that of the spinel crystals laying straight beneath them. The amount of Fe in the chemical composition of these two cases is similar (~48 wt% previously and ~51 wt% in the present study). However, the Cr/Ni ratio was different: 0.82 during a previously reported study and is currently 1.28. In the case of the present work, the internal oxidation zone is much more uniform than the one reported previously [21], where the internal oxidation zone was very much irregular and in which the tendency toward the big nodules formation was observed. TP347H steel (18%Cr, 10%Ni), oxidized under very similar conditions [26], exhibited breakaway damage, which was attributed to the slow migration of Cr and Mn ions due to the coarse grain. In the case of the material reported here (23%Cr, 18%Ni), such an effect does not occur. Despite the coarse grain, the material developed a protective chromia scale due to the internal oxidation mechanism. The oxidation resistance of the currently reported steel at 700 °C was superior in comparison to the 17%Cr-9%Ni steel at lower temperature. The latter one, after 1500 h of steam oxidation developed a two layered, thick (~110 µm) oxide scale, consisting of outer Fe oxide and an inner FeCr_2_O_4_ spinel [35], which was not foreseen for the currently tested material, since a well protecting Cr_2_O_3_ layer was formed. Fine grained, Cu-modified 304 steel at 700 °C developed the thin and protective Cr_2_O_3_ oxide scale, and the process underwent logarithmic kinetics [27] (despite the Authors’ claim that it is parabolic), whereas the coarse grain material oxidizes according to parabolic law and it developed a two-layered Fe_2_O_3_/FeCr_2_O_4_ oxide scale. What is worth mentioning is that the Cr-containing scale was not continuous. In the present study, the oxide scale is multilayered, consisting either of Fe_2_O_3_ or Cr_2_O_3_ as a main oxide scale constituent with Fe_3_O_4_ and Fe_2_NiO_4_ oxides, however, the process undergoes logarithmic kinetics. This would suggest that logarithmic kinetics is in charge while the continuous, protective chromia scale is formed, and since the oxide scale is relatively thin, the diffusion path of the ions is short. If the chromia protective layer is uneven and discontinuous, the oxidation might not be logarithmic. This is in agreement with our recently reported study, where austenitic, coarse-grained 20%Cr-25%Ni steel was investigated. The alloy, after 500 h of oxidation, formed a thick and very uneven Cr-containing layer, resulting in parabolic kinetics (with strong distortion due to spallation) [21]. The implication of this finding might be serious. If the short-time oxidation process is performed without mass gain measurement and the continuous Cr_2_O_3_ scale is formed, the Kp parameter (parabolic rate constant) should not be calculated from the oxide scale thickness, since the oxidation will probably be in the transient stage. Parabolic law will not be the controlling one yet. The good protective properties of chromia scale hinders the diffusion of metal cations and therefore keeps the corrosion process slow. If the chromia scale is discontinuous, the outward diffusion of the Fe ions is not hindered, and mass gain is accelerated since Fe oxides are growing very fast. The parabolic law will start to be in the charge and the thickness of the oxide scale will increase. The experimental results are planned to be validated with long-term TGA/oxidation tests.

The STEM investigation of the material was not affected by the oxidation process. It revealed the presence of other phases, expected due to the chemical composition of the steel.

**Figure 8:**
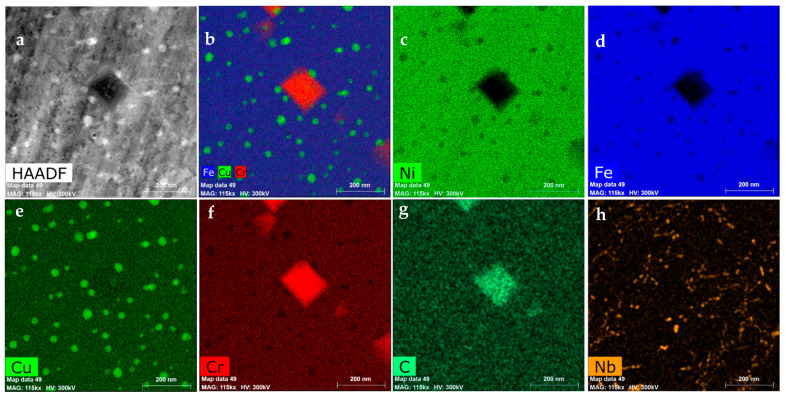
The microstructure of the 23Cr-18Ni-3Cu-1.5W-Nb-N steel is not affected by the oxidation: (**a**) The STEM-HAADF image, (**b**) The STEM-EDS compositional map of the selected elements, (**c**–**h**) The STEM-EDS map of the selected elements.

The first of them is the M_23_C_6_ phase (Figure 8a,b), nucleated not only at the grain boundaries, which are natural nucleation sites for them, but also inside the grains, over the dislocations. The indexed FFT pattern for the M_23_C_6_ precipitate present in Figure 8a is visible in Figure 7f. M_23_C_6_ precipitates are not present in the Cr-depleted zone [36] since they were dissolved to supply the Cr to form protective chromia scale. The STEM-EDS results also revealed another fine dispersed phase present in the whole volume of the alloy (Figure 8h)—NbC, evidenced by the FFT pattern in Figure 7c. Such a fine dispersed phase has a strong impact on the strength of the alloy at high temperature. A similar impact on creep properties has a Ɛ-Cu phase, precipitating at high temperature due to the addition of 3 wt.% of Cu. The presence of the latter phase, which is fully coherent with the matrix, was also confirmed in the Sanicro 25 alloy [32].

## 4. Conclusions

The performed investigations allow us to characterize oxide scale grown on 23Cr-18Ni-3Cu-1.5W-Nb-N steel. The following conclusions can be drawn:

1. Despite of the coarse grain of the investigated material, the alloy is able to develop a relatively thin and quite a uniform oxide scale.
2. The growth of the oxide scale after 500 h of oxidation can be described by the logarithmic oxidation law, which might be attributed to the formation of a continuous, protective chromia layer.
3. If mass gain is not recorded during a short oxidation period, the calculation of the Kp parameter from the oxide scale thickness might be misleading. If the oxide scale is relatively thin and continuous a chromia scale is formed, the oxidation kinetics might not be parabolic.
4. The developed oxide scale can be divided into two main areas. The first, upper one is coarse grained and consists mainly of Fe_2_O_3_, however, some crystals of Fe_2_NiO_4_ spinel are also visible. Underneath, the fine grained, internal oxidation zone is visible. It is composed of a mixture of Cr_2_O_3_ and (Fe,Mn)_2_NiO_4_.
5. Despite of the presence of Fe_2_O_3_, the developed oxide scale is protective.
6. The innermost layer of Cr_2_O_3_, is created due to the internal oxidation of Cr assuring good protection against fast oxidation.
7. The layer of the Fe_2_O_3_ undergoes spallation, however, it does not worsen the protection against oxidation.
8. In the current work, the Fe-Ni spinel was found, whereas Fe-Cr based spinels are reported elsewhere.
9. TEM investigations allowed the finding of the M_23_C_6_, Ɛ-Cu, and very fine NbC precipitates in the bulk material.
10. In order to avoid iron oxide formation, the chemical composition of the alloy might be modified. The Cr amount has to be raised when the grain size is increased.

## Figures and Tables

**Table 1:** The chemical composition of the 23Cr-18Ni-3Cu-1.5W-Nb-N steel smelted in house (in wt.%).

Fe	C	Cr	Ni	Cu	W	Mn	Nb	W	N	Si
balance	0.1	23	18	3	1.5	1	0.45	1.5	0.2	0.1

## Data Availability

The raw/processed data required to reproduce these findings cannot be shared at this time as the data also forms part of an ongoing study.
